# The cooperative difference: perceived drivers of higher care quality at home care cooperatives

**DOI:** 10.1093/haschl/qxaf118

**Published:** 2025-06-27

**Authors:** Geoffrey M Gusoff, Miguel A Cuevas, Catherine Sarkisian, Madeline R Sterling, Ariel C Avgar, Gery W Ryan

**Affiliations:** Department of Family Medicine, David Geffen School of Medicine at UCLA, Los Angeles, CA 90095, United States; Division of General Internal Medicine and Health Services Research, David Geffen School of Medicine at UCLA, Los Angeles, CA 90024, United States; Department of Medicine, David Geffen School of Medicine at UCLA, Los Angeles, CA 90095, United States; VA Greater Los Angeles Healthcare System Geriatric Research Education and Clinical Center, Los Angeles, CA 90073, United States; Department of Medicine, Weill Cornell Medicine, New York, NY 10021, United States; School of Industrial and Labor Relations, Cornell University, Ithaca, NY 14853, United States; Department of Health Systems Science, Kaiser Permanente Bernard J. Tyson School of Medicine, Pasadena, CA 91101, United States

**Keywords:** home care workers, home health aides, home care cooperatives, worker ownership, home care quality

## Abstract

**Introduction:**

The quality of care provided by home care workers (HCWs), on whom millions of Americans rely, is undermined by practices, structures, and policies that marginalize this workforce. Home care cooperatives—agencies co-owned and controlled by HCWs—represent a promising model for reducing HCW marginalization and improving care, but the specific ways in which the cooperative model may facilitate higher care quality are not well understood.

**Methods:**

We conducted 32 semistructured interviews with HCWs and other staff across 5 home care cooperatives to identify perceived drivers of improved care quality at cooperatives.

**Results:**

Respondents identified 4 main drivers of improved care quality at cooperatives: (1) increased HCW input into patient care decisions; (2) additional motivation derived from being co-owners; (3) preferential selection of high-performing, mission-driven HCWs; and (4) access to high-quality, hands-on training.

**Conclusions:**

Increasing the prevalence of these perceived quality drivers through the expansion of home care cooperatives, the adoption of cooperatives’ practices by traditional agencies, and the implementation of industry-wide policies that facilitate them may significantly improve care quality across the home care sector. However, additional research is needed to determine the role each perceived driver plays in home care quality.

## Teaser

Home care workers’ input into patient care, motivation as co-owners, selection of other invested caregivers, and access to hands-on training may be important drivers of higher care quality at home care cooperatives. Participants’ responses suggest potential agency-level practices and structures as well as industry-wide policies that may facilitate each driver to improve care quality across the home care sector.

## Introduction

With the growing number of older adults seeking to remain in their homes as they age, the demand for quality home care has never been greater.^1,2^ Home care workers (HCWs), who assist patients with activities of daily living and aspects of medical care, play a crucial role in home care quality, typically spending more time with patients than any other member of the healthcare team.^3,4^

However, HCWs are profoundly marginalized within the healthcare system, undermining their ability to provide quality care. Despite the crucial role that input from frontline care workers plays in patient safety and care outcomes, HCWs’ input is rarely elicited or acted upon by other members of the care team.^5-12^ While HCWs carry out detailed care plans across diverse home environments, they typically receive limited training with inconsistent standards.^13,14^ HCWs also face irregular hours, limited benefits, and the lowest wages in healthcare, resulting in annual turnover rates up to 82% and undermining staff consistency, an important contributor to home care quality.^15-21^

This HCW marginalization and its impacts on care quality are the result of particular practices, structures, and policies. These include agency-level practices such as the exclusion of HCWs from care planning, punitive supervisory approaches, and limited opportunities for additional training.^9,14,22,23^ Structurally, the increasing predominance of profit-driven ownership models in home care and long-term care more broadly may incentivize investor profits over long-term workforce investments and has been associated with worse care quality and patient outcomes.^24-29^ Finally, low fee-for-service reimbursement provided through Medicaid, the largest payer of HCW services, translates into low HCW compensation and ignores the value of quality HCW care in preventing costly, unnecessary hospital care.^14,30^

Within this HCW landscape, an alternative model has emerged that aims to reduce HCW marginalization and improve care quality. Home care cooperatives—agencies co-owned by HCWs themselves—enable HCWs to share in agency profits and participate in key decisions as board members and through member-wide voting.^24,31^ There are currently over 14 home care cooperatives in the US employing over 2000 HCWs, in settings ranging from the Bronx, New York to rural Wisconsin.^32^ This HCW-centering model has achieved higher wages and half the turnover rates of traditional home care agencies.^33^ Several case studies suggest cooperatives have also achieved exceptionally high levels of care quality, which may help explain their significantly higher patient retention compared to traditional agencies.^33-37^ However, the specific determinants of higher care quality at home care cooperatives have not been empirically investigated across cooperative settings.

Understanding the ways in which cooperatives may promote higher care quality can inform other agencies’ practices and structures, as well as sector-wide policies for improving HCW care quality. Therefore, we sought to elicit the perspectives of home care cooperative staff and HCWs to identify and systematically characterize perceived drivers of higher care quality at cooperatives.

## Data and methods

### Setting and study design

We conducted semistructured interviews of HCWs and office-based staff (ie, managers and schedulers) at home care cooperatives from November 2023 to June 2024. To recruit participants, we partnered with ICA Group, a nonprofit organization supporting the development of home care cooperatives across the United States.

To recruit a diverse sample at the organizational and individual level, we used a two-step sampling strategy. First, we recruited home care cooperatives across different geographies, sizes, payer types, and years in operation. ICA Group provided contact information for the cooperatives, and the principal investigator (G.M.G.) emailed them with a standard recruitment script. Participating cooperatives provided contact lists for employees across different roles (HCW vs office-based staff) and years with the cooperative. Office-based staff members, many of whom previously worked as HCWs, were included given their additional perspectives on cooperatives’ policies, practices, and care outcomes. To be eligible, participants had to be currently employed at a home care cooperative, English-speaking, and 18 years old or older. All participants received a $50 gift card and provided informed consent.

### Data collection

The semistructured interviews were conducted virtually over Zoom by the principal investigator and ranged from 45 to 60 min. The interview guide was informed by a conceptual model developed by Zarska et al., which maps the relationships between working conditions, worker outcomes, and care quality for direct care workers based on a systematic review of the literature.^18^ Drawing on this model, the interview guide asked respondents to describe specific working conditions and organizational practices at cooperatives as well as their perceived impacts on worker outcomes and quality of care. Key informants from the home care sector helped to adapt questions to the unique home care work environment, in order to elicit agency-level influences on working conditions beyond client-specific factors.

During the interviews, participants were asked about different aspects of the cooperative work environment, including their role in decision-making, care-related communication, and workplace culture. Participants were also asked whether they thought the cooperative model impacted the quality of care provided, and if so, how. In addition, they were asked about factors that may contribute to job quality and retention at cooperatives, which were analyzed in a separate analysis.^38^ Participants with experience in noncooperative caregiving settings were asked how the cooperative compared to those settings.

### Data analysis

All interviews were audio recorded, transcribed, and then coded using Dedoose software version 9.2.7. Under the supervision of a qualitative methods expert (G.W.R.), 2 investigators (G.M.G. and M.A.C.) applied a 2-level, thematic analysis coding methodology using both inductive and deductive approaches.^39-41^

In the first level of coding, we sorted responses into broad categories such as communication, motivation, and skill development, salient HCW workplace features identified in Zarska et al.'s systemic review and through key informant input. Key informant input from paid caregivers was prioritized in the development of these initial broad codes, given the study's focus on worker perceptions and since the coding investigators and other informants did not have direct experience working as paid caregivers. Through regular meetings, the 2 coding investigators compared coding decisions, resolved differences through consensus, and iteratively updated the codebook so that definitions of the broad codes better reflected general response themes within the transcripts. Additional codes were added as needed to capture broad themes that did not fit within the existing codes.

The investigators then developed codes for subthemes observed within each broad theme, meeting regularly to compare coding, iteratively refine code definitions, and address any discrepancies through consensus. The qualitative methods expert was available to adjudicate any coding discrepancies that could not be resolved by consensus and oversaw development of the codebook. Additional interviews were conducted until there was sufficient data to assess the range, salience, and variation within codes and until no new codes occurred in the data, consistent with saturation.^42^

This study was approved by our institution's Institutional Review Board.

## Results

A total of 32 participants, 23 HCWs and 9 staff members across 5 home care cooperatives, were interviewed for the study. Individual and cooperative characteristics are summarized in Table 1. Participating individuals were diverse in age, role, and tenure at the cooperative. Notably, a majority of participants (63%) reported experience working as a caregiver in another setting. The participating cooperatives varied by years in business, geography, payer, and size, including 2 small cooperatives, 1 medium-size cooperative, and 2 large cooperatives.

**Table 1.** Characteristics of participating cooperatives and individuals.

Cooperative characteristics (*n* = 5)	*n* (%)
Region	
Northeast	2 (40)
Midwest	1 (20)
Northwest	2 (40)
Cooperative size	
Small (<50 employees)	2 (40)
Medium (50-100 employees)	1 (20)
Large (>100 employees)	2 (40)
Agency tenure	
>15 years	3 (60)
7-15 years	1 (20)
0-6 years	1 (20)
Market density	
Large Urban	2 (40)
Small Urban/Suburban	2 (40)
Rural	1 (20)
Primary payer	
Medicaid	3 (60)
Private pay	2 (40)
Participant characteristics (*n* = 32)	
Gender	
Female	28 (88)
Male	4 (12)
Age	
20-29	7 (22)
30-39	7 (22)
40-49	6 (19)
50-59	3 (9)
60+	9 (28)
Role	
Home care worker	23 (72)
Staff	9 (28)
Membership status	
Worker-owner	29 (91)
Nonworker-owner	3 (9)
Worker tenure	
<2 years	13 (41)
2-10 years	13 (41)
>10 years	6 (19)
Other paid caregiving experience	
Yes	20 (63)
No	12 (38)

Our analysis revealed 4 main perceived drivers of higher care quality at cooperatives: care input, co-ownership motivation, caregiver selection, and capacity-building opportunities, summarized in Table 2.

**Table 2.** Summary of themes and subthemes identified in thematic analysis.

Theme	Description	Subthemes
Care input	Ways in which HCWs have input into how patients are cared for and how that input is taken into account in care-related decisions.	Input into the overall care plan Input related to acute patient safety issues
Co-ownership Motivation	Additional motivation to improve patient care experienced by HCWs as a result of being co-owners of the home care business.	Financial motivation Psychological motivation
Caregiver Selection	Ways in which the cooperative preferentially selects for HCWs who provide higher levels of care quality.	Active selection through interviewing and peer-vetting processes Self-selection of HCWs into cooperatives
Capacity-Building Opportunities	Formal and informal training and other HCW capacity-building opportunities provided by the cooperative directly (eg, in-house trainings) or indirectly (eg, reimbursed trainings provided by third parties).	Formal didactics Shadowing and peer mentorship opportunities

HCW, home care worker.

### Theme 1: care input

A key factor respondents perceived as improving care quality at the cooperatives was a relatively high level of HCW input into patient care decisions, which allowed office staff to respond more effectively to evolving patient needs. Several respondents attributed this level of communication to the cooperative structure itself, with 1 HCW explaining, “I own a piece of this company, so therefore my say counts, and I can go and communicate and bring back the information that may help the company and help the aides and the clients.”

One important area of HCW input was in updating the care plan, the document defining HCW tasks in relation to patients’ needs and preferences. As 1 HCW noted, “they want you to update them anytime there's any change of living or status in any way… so [the staff] is like, ‘Oh, thanks for letting me know. I'll update the care plan.’” This contrasts with other agencies that, “don't update the care plan,” and where HCW care plan suggestions, “just really weren't put through or handled,” according to 2 HCW respondents.

Another important area of HCW care input at cooperatives was around acute safety concerns. One HCW noted that cooperatives’ incorporation of HCW input prevented common safety issues around feeding, noting, “another caregiver coming in, trying to feed them solids when they potentially can asphyxiate—you don't have that.” This contrasts with another HCW's experience at a traditional agency where her warnings of a patient's fall risk were ignored, explaining, “my opinion was absolutely nothing, even though my client was in danger.”

While overall noting higher HCW care input at cooperatives than at other agencies, some respondents also noted room for improvement at large cooperatives, where care team communication could be more challenging. To that end, a staff member at a large cooperative described the creation of a “care navigator” position to enhance the role of HCW input within the care team.

### Theme 2: co-ownership motivation

Respondents noted that care quality at the cooperatives was also improved by the additional motivation HCWs have as a result of being co-owners of the business. While many HCWs explained they would aim to provide excellent care for patients even if they were not co-owners, they also noted that being co-owners of the business led them to, “go the extra mile” and “take it more seriously”, while staff described co-ownership leading to HCWs, “holding themselves to a higher standard,” and approaching patient care, “whole-heartedly and fully committed.”

For respondents, this “co-ownership motivation” to improve care was both financial and psychological. Providing high quality care was seen as an important way to increase agency profits, which HCWs shared in as co-owners. One HCW explained, “the incentive is to prosper as a whole—the more the business prospers, obviously, the more we benefit.” However, HCWs more often described the psychological aspects of this motivation. One HCW noted, “If you're an owner, you take more pride in your work, and so then you're going to want to give better care because you want to be able to give the client a good experience.” Another HCW explained, “being an owner-member, you take honor in the work that you have been given.”

### Theme 3: caregiver selection

Another factor respondents identified contributing to care quality was cooperatives’ preferential selection of mission-driven and hardworking HCWs. An HCW explained, “someone from a co-op who, like I said, is passionate about what they do, they're going to be more patient, they're going to take more time, they’re going to because they actually care,” in contrast to, as a staff member described, “somebody that's just going into this big agency to get some hours and get some health insurance.”

According to respondents, this selection occurs partly through cooperatives’ interviewing, hiring, and member confirmation processes. HCWs described a, “pretty thorough interview” and, “it wasn't just the standard interview questions.” Staff explained the importance of assessing before hiring HCWs whether, “our values are aligned with each other.” At all of the cooperatives, even hired HCWs only become full cooperative members after a probationary period and vote by other cooperative members. One HCW described this member confirmation process as, “another level of vetting” and another HCW explained, “you’re not becoming a member if you don't care about your job and the work you’re doing.” An HCW connected this relatively rigorous selection process to care quality, observing, “maybe because they’re so choosy about their employees that it's better workers and it's really helping the clients more.”

Respondents noted that mission-driven and hardworking HCWs also self-select into the cooperative model, with its mission to improve care quality and its profit-sharing structure. One HCW described this self-selection as, “we’re coming to contribute more and we’re okay to contribute more because we want this type of environment…people who make the choice to work for a co-op are choosing that because that correlates with their philosophy of how things should be, and how you know that things can be better than they traditionally are.” A staff member similarly explained, “I find that the people that find us, they're kind of searching for something different, something that's going to improve somebody else's life.” Another staff explained that hardworking HCWs particularly seek out the cooperative because, “their hard work can pay off in the form of profit-sharing.”

### Theme 4: capacity-building opportunities

Finally, respondents described capacity-building opportunities including formal training and peer mentoring, which enable them to develop skills to improve their care. Respondents noted that large cooperatives tended to provide in-house trainings, differentiated from traditional agency trainings by their length, scope, and practical nature. An HCW from a large cooperative described these trainings as “hands-on” and “really sufficient.” Another HCW contrasted this to a traditional agency where, “we only did training for 2 weeks and it wasn't hands on the way [the cooperative training] was.”

Several respondents noted that smaller cooperatives also emphasized HCW capacity-building but relied more on training opportunities through other entities such as state programs and online platforms. While some HCWs reported paying costs for initial certification training, most HCWs and staff reported that the cooperatives covered training costs. As 1 HCW noted, “they encourage a lot of continuing education as well, and even provide resources for that.” One staff member observed, “by having a pretty rigorous system for training, I think that also elevates the quality of care.” In contrast, as 1 HCW described, “with my current job that isn't [the cooperative], there was no training. They just sent us like a page thing about the client, and then you just went to their house.”

In addition to formal didactics, respondents also described unique capacity-building opportunities at cooperatives through HCW shadowing or peer mentoring. One HCW noted that shadowing while onboarding, “makes it a lot less intimidating, just going around at first with somebody who's been doing it for a while.” A staff member at a larger cooperative described a “peer mentor program” where experienced HCWs shadow new HCWs to provide guidance on patient care.

## Discussion

To our knowledge, this is the first study in which HCWs and staff across home care cooperatives were asked to identify factors contributing to care quality at cooperatives. Respondents identified HCW care input, co-ownership motivation, caregiver selection, and capacity-building opportunities as important drivers of care quality. While aspects of these factors have been described in the literature, the specific experiences of those employed by home care cooperatives provide new and important insights for future research and interventions to improve HCW care quality.

First, while care input from frontline workers has long been associated with higher care quality in hospitals and institutional settings, less is known about quality-enhancing care input practices in home care settings, where contact with coworkers and supervisors is much more limited.^10,12,43,44^ Our findings suggest particular agency-level practices, such as including HCWs in care plan development and employing roles like care navigators to facilitate HCW care input, may play an important role in improving home care quality. Furthermore, participatory agency structures, in which HCWs participate in important decisions through member-wide voting or board membership, may help facilitate HCW care-enhancing input.^8^ Future research can help clarify the roles of these practices and structures in enhancing HCW care input and ultimately care quality.

Second, employee-ownership studies suggest co-ownership motivation for high worker performance is strongest when profit-sharing is combined with participation in decision-making or a sense of “psychological ownership” among workers.^45-48^ However, to our knowledge this has not been assessed in home care, where relatively limited contact with the agency may undermine psychological ownership. We found respondents viewed both profit-sharing and psychological ownership as important motivators to improve care, with particular emphasis on the latter. This suggests that financial incentives like stock options may have a motivational role in improving HCW care quality, but they may be most impactful when combined with shared decision-making structures like HCW-majority boards and member-wide voting that promote a sense of psychological ownership.^47,48^

Third, respondents perceived that cooperatives improve care quality by preferentially selecting for mission-aligned, hardworking caregivers. These findings suggest that in the current context, in which severe HCW shortages may pressure agencies to hire whoever is willing to meet patient demand, the cooperative structure may exert a counter-pressure to thoroughly vet for quality candidates who will not only be coworkers but co-owners. According to respondents, this vetting takes place through more rigorous interview processes and HCW voting after a probationary period, practices that could be adopted by other agencies.

Our findings also suggest that the self-selection observed in other sectors, in which higher-performing workers self-select into profit-sharing firms and mission-driven workers self-select into mission-oriented organizations, may also occur at home care cooperatives.^46,49^ If this is the case, home care cooperatives’ unique combination of profit-sharing and social mission may be particularly effective at selecting for high-quality, values-aligned candidates.

Finally, respondents reported the cooperatives often provided high-quality, hands-on training, which has been associated with higher care quality in long-term care settings.^50-52^ It is possible that cooperatives provide higher quality training than traditional agencies due to increased HCW input into training materials or investments, but additional research is needed to further elucidate the relationship between the cooperative structure, training quality, and care quality. Respondents also described unique opportunities for shadowing and peer mentorship at cooperatives that contributed to quality care, training practices that could be instituted in other home care settings.

### Policy implications

In addition to agency-level practices and structures, our results also suggest potential industry-wide policies that may improve home care quality by enhancing HCW care input, co-ownership motivation, caregiver selection, or HCW capacity-building. This includes integrating HCWs more deeply into value-based care arrangements (eg, value-based payments for meeting care quality targets, shared savings from reduced hospitalizations), which could facilitate increased HCW input into the broader care team and reward high-quality caregiving. In addition, increased public investment in HCW training and national standardization of minimal training competencies could contribute to higher consistency and quality in HCW care.^14^

Finally, respondents described how the home care cooperative model—with its emphasis on co-ownership, shared decision-making, and peer-to-peer learning—appears to specifically facilitate drivers of home care quality. This suggests policies that support the expansion of home care cooperatives may also represent an important approach to improving care quality across the home care sector. This includes the development of cooperative-specific financing and technical assistance resources to overcome the barriers early cooperatives currently face in securing capital and business development support.^53^ Increased investments in these supports may enable cooperatives to develop at scale and have a significantly larger influence on care quality across the home care sector.

### Limitations

This study has several limitations. Our focus on HCWs and home care staff may overlook additional aspects of care quality more readily perceived by care recipients and their families (eg, communication with care recipients). In addition, while including only respondents from cooperatives provided a deeper understanding of cooperative-specific factors, it also introduces the potential for selection and recall bias when comparing respondents’ experiences at cooperative and noncooperative agencies. Also, non-English-speaking HCWs may have distinct experiences of caregiving and care quality not captured in this analysis of English-speaking respondents. Finally, our qualitative approach cannot assess whether, nor the extent to which, these perceived drivers actually impact care outcomes. Future studies including care recipient and family caregiver perspectives, non-English-speaking HCWs, HCWs from noncooperative settings and quantitative methods to assess the magnitude and direction of associations can provide a fuller assessment of factors impacting home care quality.

## Conclusion

Our findings suggest that HCW care input, co-ownership motivation, caregiver selection, and capacity-building opportunities may be important drivers of improved care quality at home care cooperatives. Future research can help determine the relative importance of each driver and their prevalence across home care contexts. In addition to identifying testable, potential drivers of HCW care quality, our findings also suggest potential agency-level practices and structures as well as industry-wide policies that may facilitate each driver to improve care quality across the home care sector.

## Supplementary Material

qxaf118_Supplementary_Data
